# Risk factors for stroke among anthropometric indices and lipid profiles in the Korean population: a large-scale cross-sectional study

**DOI:** 10.1038/s41598-023-29902-4

**Published:** 2023-02-20

**Authors:** Mi Hong Yim, Young Ju Jeon, Bum Ju Lee

**Affiliations:** Digital Health Research Division, Korea Institute of Oriental Medicine, 1672 Yuseong‑daero, Yuseong‑gu, Daejeon, 34054 Republic of Korea

**Keywords:** Risk factors, Epidemiology, Public health, Cardiovascular diseases

## Abstract

Stroke is strongly associated with death and disability. However, the associations between stroke and lipid profiles such as total cholesterol, triglycerides, high-density lipoprotein cholesterol (HDL-C), and red blood cells (RBCs) and anthropometric indices such as waist circumference and waist-to-height ratio (WHtR) remain unclear. The objective of this study was to investigate these relationships in a Korean population. This large-scale cross-sectional study included data from 38,190 subjects collected from 2010 to 2018 by the Korea National Health and Nutrition Examination Survey (KNHANES). Simple logistic regression models and multiple logistic regression models were used to evaluate the association of stroke with lipid profiles and anthropometric indices in the crude model, adjusted Model 1, and fully adjusted Model 2. In men, stroke was negatively associated with height, weight, and hematocrit level. Total cholesterol and triglycerides were strongly negatively associated with stroke in Model 2. Creatinine level and stroke were weakly associated. Additionally, height, weight, total cholesterol, triglycerides, and hematocrit and creatinine levels were associated with stroke both before and after adjustment. In women, in Model 2, stroke was positively associated with height, weight, and creatinine level. A strong negative association was found between total cholesterol and stroke. Stroke was negatively associated with hemoglobin level, hematocrit level, and RBCs. Additionally, total cholesterol, hemoglobin level, hematocrit level, creatinine level, and RBCs were associated with stroke both before and after adjustment. Weight and height were more closely associated with stroke than waist circumference and WHtR in Korean men. Our results suggested that the association of stroke with triglycerides, height, and weight differed according to sex and that HDL-C was not associated with stroke in people of either sex.

## Introduction

In the Global Burden of Diseases, Injuries, and Risk Factors Study (GBD) 2019, which included 369 diseases and injuries, stroke was the second leading cause of disability-adjusted life-years (DALYs) for people aged 50 or older and the third leading cause of DALYs for people of all ages^1^. In the United States, 8 million people were hospitalized for stroke from 2004 to 2018; patients are 70 years old on average, and women constitute 52% of these hospitalizations^2^. Additionally, Hispanic, White, and Black individuals account for approximately 8%, 70%, and 16.6% of stroke hospitalizations, respectively^2^. To date, the well-known risk factors for fatal or nonfatal stroke are age^3,4^, sex^4–6^, overweight or obesity^6–17^, serum high-density lipoprotein cholesterol (HDL-C)^18–21^, total cholesterol and hypercholesterolemia^3,8,19,22–24^, triglyceride levels or hypertriglyceridemia and hypotriglyceridemia^21,25–27^, hemoglobin levels^28,29^, hematocrit levels^30^, creatinine concentration^31–33^, systolic blood pressure (SBP)^34^ or hypertension^7,8^, cigarette smoking^7,8,34^, heavy alcohol intake^8,20,24^, low levels of physical activity^8,20^, left ventricular hypertrophy with preexisting ischemic heart disease^34^, diabetes^7^, atrial fibrillation and heart failure^8^, and race^20^.

Although many studies have investigated the association between lipid profiles and stroke, the association between total cholesterol levels and stroke is unclear^24^. For example, studies have argued that total cholesterol levels are not associated^35^, weakly associated^3,19,23^ or strongly associated^22,24^ with stroke. Furthermore, the best anthropometric index for assessing the risk of stroke remains controversial due to differences in sex, age, race, and nationality. For example, several studies have suggested that waist circumference (WC) is a risk factor for stroke in men but not in women^9,10^, whereas another study argued that higher WC was associated with the risk of stroke in women but not in men^6^. Additionally, several studies have reported that the best obesity index to predict stroke is WC^14,17^, the waist-to-hip ratio (WHR)^13^, or the waist-to-height ratio (WHtR)^16^. Furthermore, another study argued that body mass index (BMI), WC, and WHR were all significant risk factors for stoke, regardless of race or sex^12^. Therefore, the objective of this study was to examine the association of stroke with lipid profiles such as total cholesterol, triglycerides, hematocrit, creatinine, hemoglobin, high-density lipoprotein cholesterol (HDL-C), and red blood cells (RBCs) and anthropometric indices such as waist circumference and WHtR in a Korean population. Our findings provide evidence for variables that are risk factors for stroke in Korean men and women, informing the fields of public health and epidemiology.

## Results

### General characteristics of the subjects

Sociodemographic characteristics and clinical characteristics in the stroke group and the nonstroke group are displayed according to sex in Table 1. Of the 32,030 subjects (13,924 men and 18,106 women), 876 subjects (473 men and 403 women) reported that they had been diagnosed with stroke. The proportion of men aged 60 to 69 years was higher in the stroke group (36.66%) than in the nonstroke group (18.85%). The proportion of women aged 70 to 79 years was higher in the stroke group (41.5%) than in the nonstroke group (13.28%). Significant differences between the stroke and nonstroke groups were found in household income, number of household members, marital status, education level, employment status, drinking habits, physical activity, and stress level for both sexes. There were no differences between the stroke group and the nonstroke group in terms of smoking status for women and region for either sex.

**Table 1:** General characteristics of the stroke and nonstroke groups according to sex.

Variable	Men			Women		
	Nonstroke	Stroke	*P* value	Nonstroke	Stroke	*P* value
	N = 13,451	N = 473		N = 17,703	N = 403	
Age (years)			< .001			< .001
40–49	37.63 (0.58)	8.65 (1.89)		34.98 (0.5)	6.49 (1.58)	
50–59	33.34 (0.51)	25.44 (2.77)		32.21 (0.44)	22.11 (2.75)	
60–69	18.85 (0.37)	36.66 (2.65)		19.54 (0.35)	29.91 (2.76)	
70–79	10.18 (0.26)	29.25 (2.33)		13.28 (0.29)	41.5 (2.97)	
Region			0.15			0.603
Seoul/Gyeonggi/Incheon	48.76 (0.6)	45.35 (2.81)		48.65 (0.54)	45.01 (3.12)	
Gangwon	3.01 (0.27)	4.89 (1.38)		2.85 (0.27)	3.73 (0.89)	
Daejeon/Chungcheong/Sejong	10.61 (0.44)	9.77 (1.52)		10.17 (0.41)	10.72 (2.03)	
Gwangju/Jeolla/Jeju	11.48 (0.42)	14.3 (1.89)		11.44 (0.4)	10.52 (2.03)	
Busan/Daegu/Ulsan/Gyeongsang	26.14 (0.57)	25.69 (2.51)		26.89 (0.51)	30.01 (2.72)	
Household income			< .001			< .001
1st quintile (lowest)	15.29 (0.4)	37.09 (2.65)		20.96 (0.44)	47.04 (3.15)	
2nd quintile	18.55 (0.46)	19.66 (2.2)		19.94 (0.4)	23.53 (2.59)	
3rd quintile	20.9 (0.46)	15.03 (1.99)		18.81 (0.38)	13.71 (2.06)	
4th quintile	21.21 (0.45)	11.27 (1.77)		18.96 (0.38)	11.3 (2.02)	
5th quintile (highest)	24.05 (0.57)	16.95 (2.3)		21.33 (0.51)	4.42 (1.09)	
Number of household members			< .001			< .001
1	6.86 (0.31)	12.09 (1.92)		9.18 (0.25)	21.71 (2.4)	
2	27.27 (0.49)	41.11 (2.69)		29.59 (0.43)	41.39 (2.89)	
3	25.33 (0.46)	23.95 (2.55)		27.18 (0.42)	17.98 (2.34)	
4	29.2 (0.53)	17.13 (2.35)		23.88 (0.42)	9.23 (1.83)	
≥ 5	11.35 (0.38)	5.72 (1.31)		10.16 (0.32)	9.69 (2.12)	
Marital status			0.002			< .001
Married/living together	88.11 (0.39)	83.32 (2.29)		78.16 (0.41)	59.22 (2.86)	
Widowed/divorced/separated	7 (0.28)	12.6 (2.01)		20.1 (0.39)	39.54 (2.86)	
Never married	4.89 (0.25)	4.08 (1.22)		1.73 (0.11)	1.25 (0.62)	
Education			< .001			< .001
Elementary school or below	15.07 (0.4)	29.48 (2.45)		26.36 (0.47)	57.55 (3.31)	
Middle school	13.83 (0.38)	22.96 (2.53)		14.93 (0.34)	19.23 (2.66)	
High school	33.22 (0.54)	28.27 (2.6)		34.97 (0.49)	14.97 (2.32)	
College or above	37.88 (0.68)	19.29 (2.13)		23.74 (0.53)	8.25 (1.84)	
Employment status			< .001			< .001
Employed	81.44 (0.42)	49.21 (2.8)		53.33 (0.5)	25.61 (2.72)	
Unemployed	18.56 (0.42)	50.79 (2.8)		46.67 (0.5)	74.39 (2.72)	
Alcohol intake frequency			< .001			< .001
None in the past year	16.53 (0.39)	28.94 (2.37)		37.68 (0.45)	57.94 (2.97)	
Monthly or less	17.59 (0.39)	17.34 (2.01)		35.93 (0.43)	25.76 (2.66)	
2 to 4 times a month	24.27 (0.44)	18.89 (2.12)		16.98 (0.34)	9.76 (1.57)	
2 to 3 times a week	25.89 (0.47)	20.42 (2.29)		7.16 (0.24)	3.2 (1.03)	
4 times a week or more	15.72 (0.38)	14.41 (1.96)		2.25 (0.13)	3.33 (1.28)	
Smoking status			< .001			0.165
Daily	35.4 (0.53)	26.31 (2.51)		3.6 (0.17)	2.81 (0.79)	
Sometimes	3.57 (0.21)	3.39 (1.09)		1.08 (0.1)	0.23 (0.23)	
Quit smoking	44.45 (0.52)	56.89 (2.75)		3.81 (0.17)	5.29 (1.31)	
Never smoked	16.58 (0.38)	13.41 (1.84)		91.51 (0.27)	91.67 (1.52)	
Physical activity			< .001			< .001
None	64.72 (0.54)	74.53 (2.46)		74.04 (0.45)	85.62 (2.01)	
Once a week or more	35.28 (0.54)	25.47 (2.46)		25.96 (0.45)	14.38 (2.01)	
Stress			0.002			< .001
Severe	3.37 (0.18)	3.43 (0.92)		4.76 (0.19)	10.31 (1.86)	
Moderate	18.6 (0.4)	12.75 (1.76)		20.57 (0.36)	19.51 (2.17)	
Mild	60.73 (0.48)	60.45 (2.57)		58.61 (0.44)	46.71 (3.03)	
Slight	17.31 (0.36)	23.37 (2.15)		16.05 (0.32)	23.48 (2.33)	
Hypertension			< .001			< .001
No	74.23 (0.44)	32.51 (2.54)		75.35 (0.41)	36.71 (2.95)	
Yes	25.77 (0.44)	67.49 (2.54)		24.65 (0.41)	63.29 (2.95)	
Dyslipidemia			< .001			< .001
No	85.16 (0.35)	63.35 (2.62)		80.36 (0.35)	58.13 (3.02)	
Yes	14.84 (0.35)	36.65 (2.62)		19.64 (0.35)	41.87 (3.02)	
Diabetes			< .001			< .001
No	89.13 (0.3)	66.99 (2.54)		91.38 (0.26)	74.05 (2.72)	
Yes	10.87 (0.3)	33.01 (2.54)		8.62 (0.26)	25.95 (2.72)	

Values are presented as percentages (standard errors). *P* values were derived from Rao-Scott and chi-squared tests. Integrated weights were used in all analyses. N, number of subjects.

### Association of stroke with anthropometric indices and lipid profiles

Table 2 displays the associations of stroke with anthropometric indices and lipid profiles in men. In the crude analysis, most anthropometric indices and lipid profiles, except for BMI, hepatitis B surface antigen (HBsAg), AST, and platelets, were related to stroke. However, only some of them were related to stroke after adjusting for confounders. In Model 1, which was adjusted for age and BMI, stroke was positively associated with WHtR (OR, 1.36; 95% CI, 1.12–1.66), fasting blood glucose (FBG) (OR, 1.22; 95% CI, 1.11–1.33), creatinine level (OR, 1.09; 95% CI, 1.05–1.14), WBCs (OR, 1.14; 95% CI, 1.03–1.26) and platelets (OR, 1.14; 95% CI, 1.03–1.26); stroke was negatively associated with height (OR, 0.76; 95% CI, 0.68–0.84), weight (OR, 0.58; 95% CI, 0.47–0.72), total cholesterol (OR, 0.55; 95% CI, 0.47–0.64), triglycerides (OR, 0.82; 95% CI, 0.71–0.95), hemoglobin level (OR, 0.78; 95% CI, 0.69–0.88), hematocrit level (OR, 0.76; 95% CI, 0.68–0.86) and RBCs (OR, 0.83; 95% CI, 0.73–0.94). In Model 2, which was adjusted for the additional confounders of region, household income, number of household members, marital status, education, employment status, alcohol intake frequency, smoking status, physical activity, stress, hypertension, dyslipidemia, and diabetes, stroke was negatively associated with height (OR, 0.77; 95% CI, 0.68–0.86), weight (OR, 0.59; 95% CI, 0.47–0.74) and hematocrit level (OR, 0.86; 95% CI, 0.77–0.97). Total cholesterol and triglycerides were strongly negatively associated with stroke in Model 2 (total cholesterol, OR, 0.66; 95% CI, 0.57–0.76; triglycerides, OR, 0.75; 95% CI, 0.63–0.9). There was a weak association between creatinine level and stroke (OR, 1.05; 95% CI, 1.01–1.09) in Model 2. Additionally, height, weight, total cholesterol, triglycerides, hematocrit level, and creatinine level were associated with stroke both before and after adjustment.

**Table 2:** Association of stroke with anthropometric indices and lipid profiles in men.

Variable	Nonstroke	Stroke	Crude analysis		Model 1		Model 2	
	Mean ± SE	Mean ± SE	OR (95% CI)	*P* value	OR (95% CI)	*P* value	OR (95% CI)	*P* value
BMI (kg/m^2^)	24.35 ± 0.03	24.53 ± 0.16	1.06 (0.96–1.18)	0.229				
Height (cm)	169.28 ± 0.07	165.91 ± 0.31	0.59 (0.53–0.65)	< .001	0.76 (0.68–0.84)	< .001	0.77 (0.68–0.86)	< .001
Weight (kg)	69.9 ± 0.11	67.65 ± 0.52	0.8 (0.72–0.89)	< .001	0.58 (0.47–0.72)	< .001	0.59 (0.47–0.74)	< .001
Waist circumference (cm)	85.93 ± 0.09	87.29 ± 0.44	1.18 (1.06–1.31)	0.002	0.98 (0.79–1.22)	0.859	0.82 (0.65–1.03)	0.086
WHtR	0.51 ± 0.001	0.53 ± 0.003	1.44 (1.3–1.59)	< .001	1.36 (1.12–1.66)	0.002	1.11 (0.9–1.38)	0.334
SBP (mmHg)	122.32 ± 0.18	126.4 ± 0.86	1.27 (1.16–1.4)	< .001	1.07 (0.97–1.19)	0.183	0.99 (0.89–1.11)	0.91
DBP (mmHg)	79.8 ± 0.12	76.72 ± 0.6	0.74 (0.66–0.83)	< .001	0.93 (0.82–1.05)	0.235	1 (0.88–1.13)	0.945
Fasting blood glucose (mg/dL)	105.5 ± 0.28	114.76 ± 1.96	1.26 (1.17–1.35)	< .001	1.22 (1.11–1.33)	< .001	1.02 (0.9–1.16)	0.754
Total cholesterol (mg/dL)	192.54 ± 0.39	167.9 ± 2.27	0.48 (0.41–0.55)	< .001	0.55 (0.47–0.64)	< .001	0.66 (0.57–0.76)	< .001
HDL-C (mg/dL)	46.6 ± 0.12	44.82 ± 0.72	0.84 (0.73–0.98)	0.024	0.88 (0.75–1.02)	0.084	0.97 (0.83–1.12)	0.642
Triglycerides (mg/dL)	174.71 ± 1.73	145.16 ± 4.68	0.74 (0.65–0.86)	< .001	0.82 (0.71–0.95)	0.01	0.75 (0.63–0.9)	0.002
HBsAg	177.37 ± 10.87	88.12 ± 36.19	0.89 (0.76–1.03)	0.108	0.93 (0.81–1.07)	0.33	0.96 (0.83–1.11)	0.578
AST (IU/L)	25.7 ± 0.18	25.61 ± 0.81	0.99 (0.9–1.1)	0.918	1 (0.88–1.14)	0.999	0.98 (0.86–1.13)	0.814
ALT (IU/L)	26.61 ± 0.23	24.39 ± 0.72	0.86 (0.76–0.98)	0.022	0.97 (0.86–1.09)	0.606	0.94 (0.82–1.07)	0.349
Hemoglobin (g/dL)	15.21 ± 0.01	14.65 ± 0.09	0.67 (0.61–0.75)	< .001	0.78 (0.69–0.88)	< .001	0.89 (0.79–1)	0.059
Hematocrit (%)	45.17 ± 0.04	43.59 ± 0.23	0.67 (0.6–0.74)	< .001	0.76 (0.68–0.86)	< .001	0.86 (0.77–0.97)	0.012
BUN (mg/dL)	15.53 ± 0.05	16.47 ± 0.42	1.14 (1.04–1.24)	0.006	1.03 (0.89–1.19)	0.664	0.98 (0.86–1.12)	0.782
Creatinine (mg/dL)	0.97 ± 0.003	1.11 ± 0.08	1.09 (1.05–1.14)	< .001	1.09 (1.05–1.14)	< .001	1.05 (1.01–1.09)	0.011
WBCs (Thous/uL)	6.55 ± 0.02	6.76 ± 0.11	1.11 (1.01–1.23)	0.034	1.14 (1.03–1.26)	0.009	1.02 (0.91–1.15)	0.678
RBCs (Mil/uL)	4.86 ± 0.005	4.69 ± 0.03	0.69 (0.62–0.77)	< .001	0.83 (0.73–0.94)	0.003	0.9 (0.79–1.01)	0.078
Platelets (Thous/uL)	245.15 ± 0.6	247.9 ± 5.55	1.05 (0.89–1.23)	0.587	1.14 (1.03–1.26)	0.008	1.11 (1–1.23)	0.051

Values are presented as the means ± standard errors and odds ratios (95% confidence intervals). *P* values were derived from logistic regression analyses of men with and without adjustment. Integrated weights were used in all analyses.

Model 1: Adjusted for age and BMI.

Model 2: Adjusted for age, BMI, region, household income, number of household members, marital status, education level, employment status, alcohol intake frequency, smoking status, physical activity, stress, hypertension, dyslipidemia, and diabetes.

SE, standard error; OR, odds ratio; CI, confidence interval; WHtR, waist-to-height ratio; HDL-C, high-density lipoprotein cholesterol; HBsAg, hepatitis B surface antigen; AST, aspartate aminotransferase; ALT, alanine aminotransferase; BUN, blood urea nitrogen; WBCs, white blood cells; RBCs, red blood cells.

Table 3 presents the associations of stroke with anthropometric indices and lipid profiles in women. In the crude analysis, stroke was associated with anthropometric indices and lipid profiles, except for weight, DBP, HBsAg, ALT, WBCs and platelets. However, only some of these were associated with stroke after adjustment, as was the case for men. In Model 1, which was adjusted for age and BMI, stroke was positively associated with WC (OR, 1.3; 95% CI, 1.03–1.64), FBG (OR, 1.2; 95% CI, 1.11–1.29), triglycerides (OR, 1.09; 95% CI, 1.01–1.17) and creatinine level (OR, 1.13; 95% CI, 1.04–1.23) but negatively associated with total cholesterol (OR, 0.65; 95% CI, 0.57–0.74), hemoglobin level (OR, 0.82; 95% CI, 0.72–0.93), hematocrit level (OR, 0.82; 95% CI, 0.72–0.94) and RBCs (OR, 0.83; 95% CI, 0.73–0.96). In Model 2, which was adjusted for the same confounders as those for men, stroke was positively related to height (OR, 1.2; 95% CI, 1.01–1.42), weight (OR, 1.4; 95% CI, 1.02–1.91) and creatinine level (OR, 1.1; 95% CI, 1.02–1.19). A strong negative association was found between total cholesterol and stroke (OR, 0.73; 95% CI, 0.63–0.84) in Model 2; additionally, stroke was negatively associated with hemoglobin level (OR, 0.82; 95% CI, 0.71–0.93), hematocrit level (OR, 0.83; 95% CI, 0.72–0.96), and RBCs (OR, 0.83; 95% CI, 0.72–0.96). Total cholesterol, hemoglobin level, hematocrit level, creatinine level, and RBCs were associated with stroke both before and after adjustment.

**Table 3:** Association of stroke with anthropometric indices and lipid profiles in women.

Variable	Nonstroke	Stroke	Crude		Model 1		Model 2	
	Mean ± SE	Mean ± SE	OR (95% CI)	*P* value	OR (95% CI)	*P* value	OR (95% CI)	*P* value
BMI (kg/m^y^)	23.93 ± 0.03	24.98 ± 0.2	1.31 (1.19–1.44)	< .001				
Height (cm)	156.15 ± 0.06	154.07 ± 0.39	0.71 (0.63–0.8)	< .001	1.09 (0.94–1.26)	0.252	1.2 (1.01–1.42)	0.042
Weight (kg)	58.35 ± 0.08	59.33 ± 0.54	1.11 (1–1.24)	0.06	1.18 (0.9–1.55)	0.237	1.4 (1.02–1.91)	0.035
Waist circumference (cm)	80.21 ± 0.11	84.64 ± 0.58	1.53 (1.38–1.7)	< .001	1.3 (1.03–1.64)	0.026	1.22 (0.96–1.55)	0.102
WHtR	0.51 ± 0.001	0.55 ± 0.004	1.65 (1.49–1.83)	< .001	1.2 (0.93–1.56)	0.163	1.04 (0.78–1.39)	0.793
SBP (mmHg)	119.55 ± 0.18	128.41 ± 1.45	1.54 (1.37–1.73)	< .001	1.15 (0.98–1.35)	0.091	1.01 (0.85–1.2)	0.927
DBP (mmHg)	75.29 ± 0.1	75.66 ± 0.66	1.04 (0.91–1.19)	0.575	1.08 (0.94–1.24)	0.254	1.09 (0.93–1.26)	0.287
Fasting blood glucose (mg/dL)	99.64 ± 0.21	110.54 ± 2.01	1.29 (1.21–1.37)	< .001	1.2 (1.11–1.29)	< .001	1.06 (0.95–1.18)	0.308
Total cholesterol (mg/dL)	197.56 ± 0.34	181.96 ± 2.15	0.63 (0.55–0.72)	< .001	0.65 (0.57–0.74)	< .001	0.73 (0.63–0.84)	< .001
HDL-C (mg/dL)	52.75 ± 0.12	49.65 ± 0.69	0.76 (0.67–0.87)	< .001	0.92 (0.81–1.05)	0.243	0.95 (0.83–1.09)	0.501
Triglycerides (mg/dL)	124.3 ± 0.88	145.26 ± 5.67	1.13 (1.06–1.2)	< .001	1.09 (1.01–1.17)	0.036	1.06 (0.95–1.17)	0.296
HBsAg	152.62 ± 8.56	154.05 ± 63.86	1 (0.88–1.14)	0.982	1.03 (0.9–1.17)	0.662	1.07 (0.93–1.23)	0.322
AST (IU/L)	21.86 ± 0.09	23.61 ± 0.58	1.09 (1.03–1.16)	0.002	1.03 (0.94–1.12)	0.505	1.02 (0.93–1.12)	0.729
ALT (IU/L)	19.35 ± 0.13	20.19 ± 0.72	1.05 (0.98–1.13)	0.173	0.98 (0.86–1.11)	0.696	0.93 (0.79–1.1)	0.417
Hemoglobin (g/dL)	13.14 ± 0.01	12.98 ± 0.07	0.89 (0.8–0.99)	0.027	0.82 (0.72–0.93)	0.001	0.82 (0.71–0.93)	0.003
Hematocrit (%)	39.87 ± 0.03	39.43 ± 0.21	0.88 (0.78–0.99)	0.032	0.82 (0.72–0.94)	0.003	0.83 (0.72–0.96)	0.011
BUN (mg/dL)	14.44 ± 0.04	16.41 ± 0.42	1.36 (1.25–1.47)	< .001	1.14 (1–1.31)	0.055	1.14 (0.99–1.3)	0.069
Creatinine (mg/dL)	0.72 ± 0.002	0.81 ± 0.03	1.16 (1.08–1.25)	< .001	1.13 (1.04–1.23)	0.004	1.1 (1.02–1.19)	0.013
WBCs (Thous/uL)	5.79 ± 0.02	5.95 ± 0.09	1.1 (0.99–1.22)	0.065	1 (0.89–1.13)	0.945	0.9 (0.78–1.03)	0.134
RBCs (Mil/uL)	4.35 ± 0.003	4.26 ± 0.02	0.78 (0.68–0.89)	< .001	0.83 (0.73–0.96)	0.01	0.83 (0.72–0.96)	0.012
Platelets (Thous/uL)	262.31 ± 0.58	255.21 ± 3.61	0.89 (0.79–1)	0.059	0.95 (0.85–1.07)	0.379	0.93 (0.82–1.06)	0.268

Values are presented as the means ± standard errors and odds ratios (95% confidence intervals). *P* values were derived from logistic regression analyses of women with and without adjustment. Integrated weights were used in all analyses.

Model 1: Adjusted for age and BMI.

Model 2: Adjusted for age, BMI, region, household income, number of household members, marital status, education level, employment status, alcohol intake frequency, smoking status, physical activity, stress, hypertension, dyslipidemia, and diabetes.

SE, standard error; OR, odds ratio; CI, confidence interval; WHtR, waist-to-height ratio; HDL-C, high-density lipoprotein cholesterol; HBsAg, hepatitis B surface antigen; AST, aspartate aminotransferase; ALT, alanine aminotransferase; BUN, blood urea nitrogen; WBCs, white blood cells; RBCs, red blood cells.

For both sexes, height, weight, total cholesterol, hematocrit level, and creatinine level were associated with stroke in the fully adjusted model (Model 2). Men and women differed in that triglycerides were only related to stroke in men and hemoglobin levels and RBCs were only related to stroke in women, according to Model 2.

## Discussion

The association between anthropometric indices (obesity) and the risk of stroke, including the best index to predict stroke, is unclear due to differences in the age, sex, race, and nationality of individuals. In this study, we applied a fully adjusted model and found the following: (1) height, weight, hematocrit level, total cholesterol, triglycerides, creatinine level, and hematocrit level were associated with stroke in men; and (2) stroke was related to height, weight, creatinine level, total cholesterol, hemoglobin level, hematocrit level, and RBCs in women.

Studies investigating anthropometric indices as risk factors for stroke have yielded controversial results. For example, Hu et al.^9^ examined the association of several adiposity indices with ischemic stroke by following a cohort of Finnish patients and reported that WC was a risk factor for ischemic stroke only in men but that BMI was a risk factor for ischemic stroke in people of both sexes. Dey et al.^10^ investigated the relationships of WC and BMI with stroke in 70-year-old men and women with a 15-year follow-up in Sweden and reported that high WC and BMI were risk factors for stroke in men but not in women. Additionally, Cong et al.^11^ tested the association of a combination of BMI and WC with the risk of stroke in a large-scale cohort study in China. They argued that anthropometric patterns related to the risk for stroke were predicted by this combination. However, Furukawa et al.^6^ assessed the associations of WC and BMI with the risks of stroke and myocardial infarction in a follow-up cohort study in an urban Japanese population and reported that high WC was associated with the risk of CVD and stroke in women but not in men. Yatsuya et al.^12^ examined differences in the relationship of anthropometric indices with ischemic stroke according to sex as well as ethnicity in a follow-up cohort study in the US. They reported that Black individuals had a higher incidence of stroke than White individuals but that obesity indices (such as BMI, WC, and WHR) were significant risk factors for stroke regardless of sex or ethnicity. Walker et al.^13^ predicted stroke using BMI and the WHR in a follow-up cohort study of male US health professionals and argued that higher WC and WHR, but not elevated BMI, were predictors of stroke. Similarly, Cho et al.^14^ tested the association between WC and the risk of myocardial infarction and ischemic stroke using the National Health Insurance Service data of Korean men and women. They reported that WC had a significant linear relationship with the risks of these diseases and that WC was a better predictor than BMI. In contrast, Saito et al.^15^ assessed the relationship of BMI and weight change with incident stroke in a follow-up study in Japan and reported that higher BMI in women was related to an increased risk of stroke but that in men, the relationship was weak. Moreover, Xu et al.^16^ explored the relationships of WHtR, BMI, and WC with ischemic stroke in a population-based cohort study among Mongolian men in northern China. They suggested that WHtR was more likely to predict stroke than BMI and WC. Winter et al.^17^ examined the associations of WC and BMI with stroke and transient ischemic attacks in a case–control study in Germany and reported that WC was associated with the risk of stroke and transient ischemic attacks, regardless of other vascular risk factors, and was a better predictor of both diseases than BMI. Furthermore, the association between stroke and obesity differs according to sex^5,6^. For example, Rodríguez-Campello et al.^5^ evaluated sex differences in obesity indices for the risk of ischemic stroke in a case–control study in Spain and reported that WC was associated with stroke in women but that in men, BMI was negatively associated with the risk of stroke. Our findings differed from the results of previous studies, which argued that WHtR, BMI, WHR, or WC were more closely associated with the risk of stroke than the others. We found that height and weight were more closely related to stroke than WC and WHtR in both sexes in the fully adjusted model. One explanation of this phenomenon may be the differences in the variables or confounders included in models as well as differences in the race, age, sex, nationality, and sociodemographic characteristics of participants. In the fully adjusted model, we found that WC and WHtR were not associated with stroke in individuals of either sex, but in all models (adjusted and unadjusted), height and weight predicted stroke in men.

The association between lipid profiles, including total cholesterol, and stroke is unclear due to differences in age, sex, and ethnicity among individuals. To determine the association between total cholesterol levels and stroke, a prospective study collaboration^35^ reviewed 45 prospective observational cohort studies that included 450,000 subjects with 3–30 years of follow-up. The collaboration reported that total cholesterol was not associated with stroke after adjusting for study, sex, age, diastolic blood pressure (DBP), history of heart disease, and ethnicity. However, some studies reported a weak association between total cholesterol and stroke. Lewington et al.^3^ performed a meta-analysis that included 61 prospective observational studies conducted mainly in Europe or America and covering 900,000 subjects; they reported that total cholesterol was weakly associated with total stroke mortality in individuals who were 40–59 years old. Similarly, Peters et al.^23^ investigated the association of total cholesterol with cardiovascular disease and total stroke with a meta-analysis of 97 cohorts with 1,022,276 total subjects; they argued that total cholesterol had a weak effect on the total stroke risk in both men and women. Furthermore, several studies have suggested a strong association between total cholesterol and stroke. Zhang et al.^24^ examined the relationship between total cholesterol and stroke with a meta-analysis of 29 prospective cohort studies in the Asia–Pacific region. They reported that total cholesterol levels were strongly related to the risk of fatal and nonfatal ischemic stroke and weakly associated with the risk of fatal hemorrhagic stroke. Additionally, Cui et al.^22^ examined total cholesterol levels and ischemic stroke with a 12-year follow-up in a Japanese population and reported that elevated total cholesterol levels were a risk factor for ischemic stroke in Japanese men.

Studies have also investigated the levels of other lipids and their association with the risk of stroke. Sacco et al.^18^ investigated the relationship between HDL-C and ischemic stroke in an ethnically diverse population in the US with a case–control study. They reported that elevated HDL-C levels were related to a decreased risk of ischemic stroke in elderly individuals and in individuals of different ethnicities and argued that HDL-C is a significant and modifiable risk factor for stroke. Interestingly, Wannamethee et al.^19^ examined the association of serum total cholesterol and HDL-C with the risk of stroke in middle-aged British men in a prospective study and reported that increased total cholesterol levels were weakly positively associated with nonfatal stroke, but high HDL-C levels were associated with a significant decrease in the risk of nonfatal stroke. Dziedzic et al.^26^ evaluated the association between serum triglycerides and stroke severity on admission in Scandinavian individuals and suggested that subjects who experienced severe stroke showed lower serum triglyceride levels than those who experienced mild or moderate stroke. In contrast, Lee et al.^21^ examined the relationships of triglycerides and HDL-C with stroke and coronary heart disease in a prospective cohort of American Indians and reported that high triglyceride and low HDL-C levels were associated with an increased risk of ischemic stroke. Similarly, Bang et al.^27^ evaluated the association between the serum lipid panel and the occurrence of atherosclerotic stroke and argued that high triglyceride levels and nonhigh-density lipoprotein, but not LDL-C, were related to an increased risk of large-artery atherosclerotic stroke. Interestingly, Choi et al.^25^ examined the relationship between serum triglyceride levels and acute ischemic stroke and reported that both hypertriglyceridemia and hypotriglyceridemia were risk factors for poor outcomes in ischemic stroke. Turning to blood-related indices, Barlas et al.^28^ investigated the association between hemoglobin levels and anemia in stroke mortality in the UK Regional Stroke Register with a cohort study and suggested that increased hemoglobin levels were related to increased mortality within the first month and that subjects with anemia experienced elevated mortality with stroke. Yang et al.^30^ explored the association between hematocrit levels and the incidence of stroke in a Chinese population with a longitudinal cohort study and demonstrated that higher hematocrit levels were related to a higher incidence of stroke, mainly ischemic stroke. Additionally, Panwar et al.^29^ assessed the association between hemoglobin levels and stroke according to sociodemographic or clinical factors in Black and White adults in the US with a cohort study. They reported that higher and lower hemoglobin levels in women (but not men) were related to a higher risk of incident stroke. Wannamethee et al.^31^ examined the association between serum creatinine concentration and the risk of stroke events and all-cause mortality in middle-aged men in 24 British towns with a follow-up study. They reported that increased creatinine concentrations were related to a significant increase in the risk of stroke in both healthy and hypertensive men. Friedman^32^ evaluated the association between creatinine levels and survival among patients admitted for stroke and reported that serum creatinine levels were an independent predictor of survival after stroke. In addition, He et al.^33^ tested the relationship between the albumin-to-creatinine ratio and the risk of first stroke in Chinese subjects with hypertension in a follow-up study and reported that hypertensive subjects with an albumin-to-creatinine ratio ≥ 10 mg/g showed a significantly higher risk of first ischemic stroke or first stroke. Our findings were similar to the results of previous studies that suggested that total cholesterol is strongly associated with stroke^22,24^ or weakly associated with stroke^3,23^. However, our findings were inconsistent with those of previous studies^18,19,21,27^ that suggested that HDL-C was related to stroke. We did not find that HDL-C was associated with stroke in either men or women in the adjusted or fully adjusted models; an association was found only in the crude models. Additionally, our findings were consistent with the results of previous studies indicating that triglyceride levels are related to stroke in men and women^21,25–27^, except that we found this association in only women in the fully adjusted model. Additionally, our results were consistent with previous results showing that creatinine levels were related to stroke^32,33^, as we found that the creatinine level was associated with the disease in both men and women.

Regarding the pathophysiological aspect of the association between stroke and body height, for a long time, negative or inverse associations between body height and stroke in various ethnic groups, countries, and in both sexes have been reported^36–41^. For example, Njølstad et al.^37^ assessed height as a risk factor for stroke in a 15-year follow-up study of 13,266 adults in Norway and reported that height was negatively associated with stroke and that an increase of 5 cm in height decreased the aged-adjusted risk of stroke by 25% in women and 18% in men in a dose-dependent manner. In view of pathophysiological or physiological aspects of this negative association between stroke and body height, a short height itself may increase the risk of stroke, and body height is negatively related to heart rate. Short height may affect total, hemorrhagic, and ischemic strokes by pathophysiologic mechanisms^36–38,42^. Additionally, height was associated with vessel diameter and diastolic dysfunction^36,42–44^ and was negatively associated with central arterial pressure augmentation due to a shorter distance to positions of peripheral pulse wave reflection^45^. Short people suffer from cardiac overload and diastolic dysfunction because they experience a greater central pressure augmentation^42,46^. In another physiological explanation, tall subjects have greater lung capacity and higher pulmonary function and independently defend against cardiovascular disease^36,44,47^. Additionally, insulin-like growth factor-I (IGF-I) may play a role in the mechanism of association between height and cardiovascular disease^36,44^. Insulin resistance related to subclinical inflammation informs the development of cardiovascular disease and diabetes^48^. Insulin resistance is lower in tall subjects than in short subjects, and a decrease in insulin resistance in tall subjects may defend against cardiovascular disease^36,49^. Therefore, these pathological functions explain the higher prevalence among short people.

Regarding pathophysiological explanations for the association between body weight and stroke, obesity is generally considered a common risk factor for stroke. However, recently, several studies have demonstrated that prevalence, recurrent stroke, and mortality of stroke were higher among underweight than among normal or obese subjects^50–53^. For example, Rodríguez-Castro^53^ compared clinical evolution and inflammatory balance between normal and obese subjects after ischemic stroke and reported that obese subjects showed better neurological impairment recovery and did not show worse clinical evolution than normal subjects after stroke. In pathophysiological mechanisms, obesity may balance the inflammatory reaction by an anti-inflammatory flow reinforced in the first stroke^52^. Lean body mass is quickly lost after stroke, and loss of bone mineral content is difficult to prevent^54^. The loss of body muscle mass after stroke causes weight loss, even though fat mass increases^51,55^. Generally, patients after stroke had a weight loss greater than 3 kg after 4 months^56^. The mechanisms of weight loss after stroke were attributed to inflammation, impaired glucose metabolism, eating difficulties, denervation, remodeling, hemorrhagic stroke, disuse, low prealbumin level, spasticity and a combination of these factors or others^51,55,56^. Adipocytes synthesize active molecules such as adipokines. Adipokines may play a role in protecting the myocardium, while adipose tissue causes diastolic dysfunction^50,57^. To date, some studies have suggested the “obesity paradox” or “lean paradox”, but this issue is still controversial.

This study has several limitations. We could not establish cause-effect relationships due to its retrospective cross-sectional nature. Additionally, we did not consider stroke subtypes because the data used in this study did not provide the subtypes. Therefore, further study is needed on the independent effect of risk factors according to stroke subtypes. Additionally, our findings did not include information on drug intake because drugs vary. Further study is needed to consider a greater number of confounders, such as various medications used by subjects to treat stroke. Finally, there was limited accurate diagnosis information in the questionnaires because these data were collected through health interviews.

Despite these limitations, the statistical results in the present study are strong because the KNHANES represents a nationally representative sample of the very large Korean population. Additionally, we evaluated a wide range of variables from anthropometric and blood profiles for men and women.

In conclusion, we examined the association of stroke with anthropometric indices and lipid profiles in a Korean population. For both men and women, height, weight, total cholesterol, hematocrit level, and creatinine level were associated with stroke in the fully adjusted model. The sexes differed in that triglycerides were related to stroke in men, whereas hemoglobin levels and RBCs were associated with stroke in women. Our findings provide important information on risk factors for stroke among Korean men and women that can benefit public health and epidemiology. However, the best indicator of stroke among various anthropometric indices is controversial, and the association between stroke and lipid profiles is unclear. Further study is needed to confirm the best predictor of stroke among various anthropometric indices and lipid profiles.

## Methods

### Data source and study subjects

This study was based on data collected from 2010 to 2018 by the Korea National Health and Nutrition Examination Survey (KNHANES), which is a nationwide cross-sectional survey performed by the Korea Centers for Disease Control and Prevention (KCDC). The KNHANES consisted of a health examination, health interview, and nutrition survey and was designed using a complex, three-stage clustered sampling method to represent the entire Korean population^58,59^. A detailed description of the KNHANES and the data are available on its official website (https://knhanes.kdca.go.kr/). Anyone can freely access the data without any administrative permissions (https://knhanes.cdc.go.kr/knhanes/main.do and http://www.kdca.go.kr/). All data provided by the KNHANES are anonymized, and therefore, the data used in this study were anonymized.

**Figure 1:**
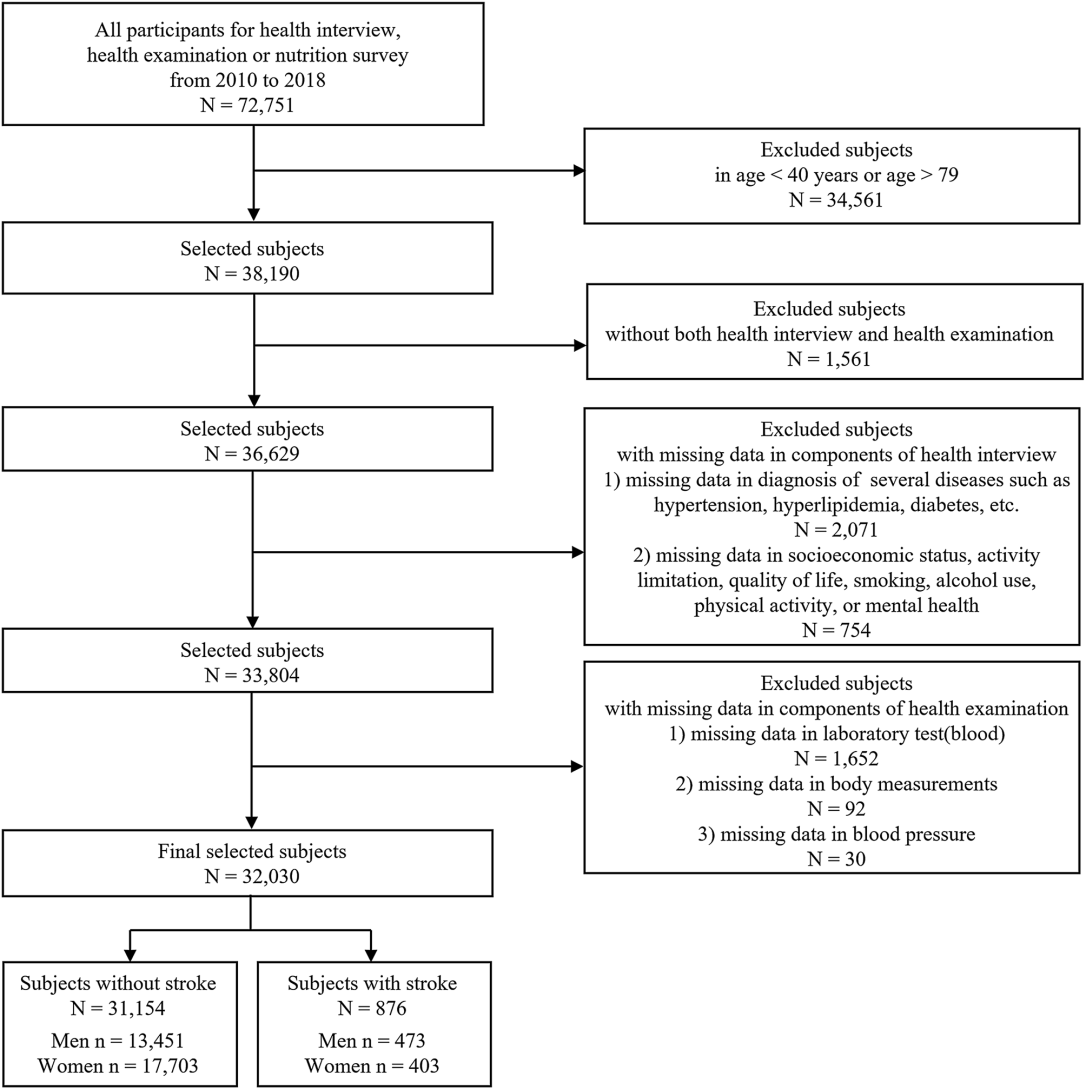
Flowchart of the study participant selection process.

A total of 72,751 subjects completed health interviews, health examinations, and nutrition surveys in the KNHANES V-VII from 2010 to 2018. Among them, we selected 38,190 subjects aged 40 to 79 and then excluded 6,160 subjects with missing data on the diagnosis of stroke, laboratory tests (blood), anthropometric measurements, blood pressure, socioeconomic status, smoking status, alcohol use, physical activity, mental health, etc. Finally, 32,030 subjects (13,924 men, 18,106 women) were included in this study. Figure 1 shows the detailed inclusion and exclusion criteria and the number of subjects.

All subjects who participated in this survey signed informed consent forms, and the KNHANES V-VII 2010–2018 protocols were approved by the Institutional Review Board of the KCDC (2010-02CON-21-C, 2011-02CON-06-C, 2012-01EXP-01-2C, 2013-07CON-03-4C, 2013-12EXP-03-5C, 2018-01-03-P-A). This study obtained approval for an exemption from the Institutional Review Board of the Korea Institute of Oriental Medicine (I-2109/008-001). The KNHANES was conducted in accordance with the Declaration of Helsinki and was approved by the Korean Ministry of Health and Welfare. All methods were performed in accordance with relevant guidelines and regulations.

### Definition of stroke

Information on stroke was obtained using a health interview survey. To avoid respondent recall bias regarding the diagnosis of stroke, the health interview survey was conducted during a face-to-face health interview by well-trained staff according to the guidelines for each item. Subjects who answered “yes” to the question “Have you ever been diagnosed with a stroke by a physician?” were placed in the stroke group. Subjects who answered this question with “no” were placed in the nonstroke group.

### Sociodemographic status, health behaviors, anthropometric measurements, and blood tests

Information on the sociodemographic status and health behaviors of participants was collected in the health interview. Anthropometric measurements and blood tests were obtained by a health examination. The health interview was performed using a self-administered questionnaire or face-to-face interview. The health examination was conducted by well-trained medical staff according to standardized protocols using equipment that was regularly calibrated.

Participants were classified into four groups based on age according to decade (40–49 years old, 50–59 years old, 60–69 years old, and 70–79 years old). The regions were classified as Seoul/Gyeonggi/Incheon, Gangwon, Daejeon/Chungcheong/Sejong, Gwangju/Jeolla/Jeju, and Busan/Daegu/Ulsan/Gyeongsang. Household income was classified into quintiles from the 1st quintile (lowest) to the 5th quintile (highest). The number of household members was classified as 1, 2, 3, 4, and 5 and over. Marital status was classified as married/living together, widowed/divorced/separated, and never married. Education was classified as elementary school or below, middle school, high school, and college or above. Employment was dichotomized into unemployed and employed. Drinking habits were classified into five groups according to the frequency of alcohol consumption: not at all for the past year, monthly or less, 2 to 4 times a month, 2 to 3 times a week, and 4 times a week or more. Smoking status was classified into four groups: smoking every day, smoking sometimes, quit smoking, and never smoked. Physical activity was dichotomized based on regularity. Stress level was classified as severe, moderate, mild, and slight according to the answer to the question about how much stress an individual usually felt in his or her daily life. Conditions such as hypertension, dyslipidemia, and diabetes were dichotomized depending on whether a subject had ever been diagnosed with the condition by a physician.

The height, weight, and WC of participants were measured while they wore light clothes to the nearest 0.1 cm (Seca 225; Saca, Hamburg, Germany), 0.1 kg (GL-6000–20; G-tech, Uijeongbu-si, Korea), and 0.1 cm (Seca 200; Saca, Hamburg, Germany), respectively. Height was measured with all four parts of the heel, buttocks, back, and back of the head touching the vertical board after removing accessories such as hats, hairpins and hair ties, loosening the hair, and taking off shoes and socks^60–62^. Weight was measured without personal belongings such as glasses, mobile phones, accessories, and locker keys after taking off the shoes and socks^60–62^. WC was measured horizontally across the middle between the lower part of the last rib and the upper part of the iliac crest on the side after lifting the clothes over the waist to expose bare skin^59,62^. Body mass index (BMI) and WHtR were calculated as weight divided by height squared (kg/m^2^) and WC divided by height, respectively. Blood pressure was measured three times in a comfortable sitting position using a mercury sphygmomanometer (Baumanometer Wall Unit 33 (0850); Baum Inc., Copiague, NY, USA). After resting comfortably for 5 min, blood pressure was measured at a point 3 cm above the elbow crease of the right arm with the participants leaning back against the back of a chair and keeping the spine in a straight line^60–62^. The average of the second and third measurements was used as the final blood pressure. Blood samples were collected from the cephalic vein or median cubital vein of the subject after a minimum fasting period of 8 h and analyzed using automatic analyzers such as a Hitachi Automatic Analyzer 7600 (Hitachi Co., Ltd., Tokyo, Japan) or XE-2100D (Sysmex Corp., Kobe, Japan) to obtain indices such as fasting blood glucose (FBG), total cholesterol, HDL-C, triglycerides, hepatitis B surface antigen (HBsAg), aspartate aminotransferase (AST), alanine aminotransferase (ALT), hemoglobin level, hematocrit level, blood urea nitrogen (BUN), creatinine level, white blood cells (WBCs), red blood cells (RBCs), and platelets.

### Statistical analysis

All statistical analyses were performed reflecting the complex sample design (two-stage stratified cluster sampling) based on the guidelines provided by the KCDC. The sampling weights, sampling units, and strata of these guidelines were used in calculating all statistics in this study to represent the Korean population. We conducted all statistical analyses using the complex samples procedure in SPSS Statistics, version 23.0 (IBM Corp., Armonk, NY, USA) and applied a significance level of 0.05.

General linear model analyses for continuous variables and Rao-Scott chi-squared tests for categorical variables were conducted to assess the differences in general characteristics between the stroke and nonstroke groups for each sex. The results are indicated as the means ± standard errors (SEs) for continuous variables and percentages (SEs) for categorical variables. Simple logistic regression models and multiple logistic regression models were used to evaluate the association of stroke with lipid profiles and anthropometric indices, depending on whether covariates were included after standardization of the data for each sex. Age and BMI were designated as covariates in the first adjusted logistic regression analysis (Model 1). The confounders of age, BMI, region, household income, number of household members, marital status, education level, employment status, alcohol intake frequency, smoking status, physical activity, stress, hypertension, dyslipidemia and diabetes were designated as covariates in the second adjusted logistic regression analysis (Model 2). The results are presented as odds ratios (ORs) with 95% confidence intervals (CIs) and p values.

## Data Availability

Data used in the present study are available from the Korea National Health and Nutrition Examination Survey (KNHANES), which is a nationwide cross-sectional survey performed by the Korea Centers for Disease Control and Prevention (KCDC). Anyone can freely access the data (https://knhanes.cdc.go.kr/knhanes/main.do and http://www.kdca.go.kr/).
